# Microplastics in Certain Commercial Finfish and Shellfish From Cox's Bazar Fish Landing Center, Bangladesh: Evaluating Abundance and Risks

**DOI:** 10.1155/sci5/9515482

**Published:** 2025-07-07

**Authors:** Md. Shahriar Ahmed, Md. Khalid Saifullah, Mst. Afifa Khatun, Ananya Chakraborty, Anika Tasnim, Upayan Anam, Mohammad Toha, Md. Kamruzzaman Munshi, Mohammad Amzad Hossain, Mohammed Mahbub Iqbal

**Affiliations:** ^1^Laboratory of Aquatic Biodiversity and Ecophysiology, Department of Fish Biology and Genetics, Sylhet Agricultural University, Sylhet 3100, Bangladesh; ^2^Department of Aquatic Resource Management, Sylhet Agricultural University, Sylhet 3100, Bangladesh; ^3^Food Safety and Quality Analysis Division, Institute of Food and Radiation Biology, Atomic Energy Research Establishment, Bangladesh Atomic Energy Commission, Dhaka, Bangladesh; ^4^Department of Environmental Science, Bangladesh University of Professionals, Mirpur, Cantonment, Dhaka 1216, Bangladesh

**Keywords:** ATR-FTIR, ecological risk, gill, GIT, risk indices

## Abstract

Microplastics (MPs) are significant environmental pollutants that have rapidly garnered public attention due to their widespread presence and harmful effects on ecosystems and human health. While MP pollution in the coastal regions has been widely reported, their potential impacts on public health are still not fully understood. The current study examined MP contamination in nine commercially important fish and shellfish species collected from the coastal waters of Bangladesh, specifically from the Bay of Bengal. MP abundances (fiber, fragment, and microbeads) were evaluated in the gastrointestinal tract (GIT) and gills of fish and in the whole body of shellfish. Fibers were the most prevalent MP types found across the samples. In the case of gills, the highest abundance of MPs was found in Bombay duck, while the lowest was in pomfret. For GIT samples, hilsa showed the highest MP concentration, whereas the lowest was observed in bombay duck. Moreover, the highest level was observed in crabs, while the lowest was in squid (*p* < 0.05), likely because crabs are benthic feeders exposed to sediment-bound MPs, whereas squids are pelagic predators with lower exposure and more selective diets. Attenuated total reflectance Fourier transform infrared research revealed that the major polymer types were polymethyl methacrylate (43.33%), ethylene vinyl acetate (23.33%), nitrile butadiene rubber (1.67%), polypropylene (5%), polycarbonate (3.33%), acrylonitrile butadiene styrene (6.67%), nylon (5%), high-density polyethylene (1.67%), polyvinyl chloride (6.67%), and polyurethane (3.33%). MP contamination in fish and shellfish was assessed using contamination factor and pollution load index values, both below 10, indicating low to moderate pollution levels. The polymer hazard index further categorized the identified polymer types into risk levels ranging from low to very high, highlighting potential ecological and health concerns. These results underscore the urgent need for effective environmental management and continuous monitoring to mitigate MP-related risks.

## 1. Introduction

In developing nations across Asia, including Bangladesh, the proliferation of plastic pollution is intensifying due to a combination of factors, including high population density, rapid urbanization, industrial development, and inadequate waste management infrastructure, leading to environmental degradation and public health risks [1, 2]. Plastics, valued for their low cost, flexibility, and durability, contribute to this contamination through primary microplastics (MPs) from industrial and consumer products and secondary MPs generated by the degradation of larger plastic waste [3, 4]. The commercial production of plastics was initiated in the 1950s and has increased by approximately 9% per year worldwide [2, 5]. In 2022, global plastic production reached 400.3 million metric tons (Mt), yet only 9.4% was recycled through mechanical and chemical processes, while the majority was disposed of in landfills or released into the natural environment [6]. If the existing trends in plastic production and inadequate waste management persist, it is projected that over 12,000 Mt of plastic waste will accumulate in landfills or the natural environment by 2050 [7, 8]. Improper disposal of plastics from land-based sources, such as industrial waste, urban runoff, and degraded debris, contributes up to 80% of marine plastic pollution [9]. Additionally, Bangladesh's extensive river system makes it easier for plastic garbage to be transported, and it eventually finds its way to the sea and coastal regions via a variety of channels, such as wind, water runoff, rainfall, and flooding [10].

MPs, which are tiny particles under 5 mm in diameter, are detrimental to natural ecosystems and include both primary and secondary plastics [11]. It is classified as primary, including microbeads, pellets, and fibers, or secondary, formed by the breakdown of larger plastics through mechanical, photo-oxidative, and biological degradation [12, 13]. Biofouling can also alter the form, size, and density of MPs, potentially affecting their buoyancy [14, 15]. Significant concentrations of MPs have been detected across diverse compartments of the global marine ecosystem, including seawater, coastal beaches, ocean sediments, estuaries, seashores, mudflats, and remote islands [16, 17]. Nowadays, MPs have been found in a wide variety of marine and coastal creatures around the world, such as herbivores, carnivores, subordinate carnivores, and tertiary fish [10, 18], crustaceans [19], zooplankton [20], corals [21], and worms [22]. The digestive systems of many fish species have been well documented to contain enormous MPs [23, 24]. Ingestion of MPs by aquatic organisms has been associated with adverse effects such as digestive blockage, reduced growth, reproductive impairment, oxidative stress, immune disruption, and altered feeding behavior, impacting both short- and long-term health [25]. Humans may experience oxidative stress, cytotoxicity, neurotoxicity, immune response disturbance, cell damage, and transfer to other tissues after being exposed to MPs [26]. MPs may also act as carriers of other harmful substances, including polybrominated diphenyl ethers, heavy metals, and polycyclic aromatic hydrocarbons [27]. However, the risks of MP pollution on ecosystem services and consumers can be achieved by evaluating the rate of MP ingestion by the various aquatic biota, as commercially exploited fish serve as the primary pathway for MP transfer from the ecosystem to humans [28]. MPs have been associated with various potential health hazards in humans, such as reproductive and developmental toxicity, allergic reactions, cancer risk, immune system impairment, respiratory disorders, inflammation, oxidative stress, cell damage, infections, antimicrobial resistance, and disruption of gut microbiota [29].

Coastal regions are essential for the livelihoods and well-being of millions of people globally, where about 21 world megacities are located on the coast, and their residents benefit directly from them as well as have an impact on the environment and coastal ecosystems [30]. The northern Bay of Bengal, a crucial region for fisheries, biodiversity, and marine activities such as shipping, tourism, and transportation, hosts extensive estuarine networks and a wide range of marine habitats [31]. However, due to human activities that produce a lot of plastic, such as commercial, residential, agricultural, and industrial ones, coastal and marine habitats are already being overused and swiftly disappearing [32]. Numerous studies have demonstrated the frequency of various plastic sizes and types in shellfish and finfish throughout the Bay of Bengal's Cox's Bazar and Chittagong coastal areas [33]. MP pollution has emerged as a pervasive threat to marine ecosystems, with studies confirming its presence in over 49% of fish species globally [34]. However, there remains a significant research gap in localized assessments, particularly in South Asian coastal regions like Cox's Bazar, Bangladesh—a major hub for seafood production and consumption [35]. While global syntheses have highlighted the ingestion of MPs by marine organisms and the associated ecological and health risks, data from Bangladesh's commercially important species, such as hilsa, mackerel, and brown shrimp, are subject to changes due to increased MP pollution. This study aims to address this gap by quantifying MP abundance in selected finfish and shellfish species from Cox's Bazar and evaluating the potential risks to both marine life and human consumers. Given the increasing evidence of MP accumulation in seafood and its potential to transfer toxic substances through the food chain [36], this research is crucial for informing national food safety policies and sustainable fisheries management. Furthermore, it will enhance the global understanding of MP distribution in tropical marine environments, where data remain sparse despite high vulnerability due to dense coastal populations and inadequate waste management systems. Additionally, this research assesses the potential health risks to human health through dietary exposure and identifies the hazardous polymer by employing advanced Fourier transform infrared (FTIR) tools.

## 2. Materials and Methods

### 2.1. Study Area

**Figure 1:**
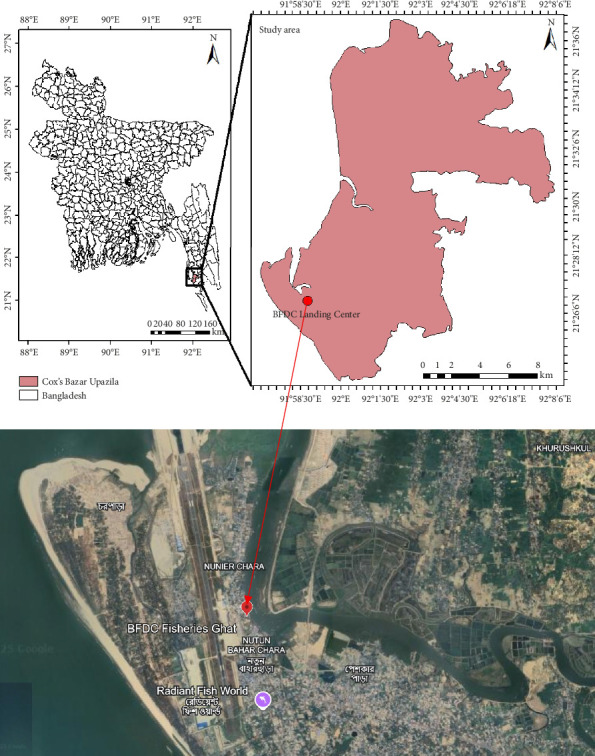
Location of fish landing center at Fishery Ghat (placemark), Cox's Bazar, Bangladesh (extracted from Google Earth map, attributed to Airbus Data SIO, NOAA, U.S. Navy, NGA, GEBCO).

Sample collection occurred in February 2023 at the Bangladesh Fisheries Development Corporation (BFDC) Landing Center in the southeastern region of Cox's Bazar (21°34′59.99″N, 92°00′60.00″E), Bangladesh. The geographic location of the sampling site is shown in Figure 1. The BFDC in Cox's Bazar is an important hub for marine biodiversity, primarily sourcing fish from the Bay of Bengal [37]. This region is home to a variety of fish species, reflecting its ecological richness. Previous studies documented a total of 54 species, comprising 42 marine fish, 7 shellfish, and 5 large fish species, including sharks and rays [38].

### 2.2. Sample Collection and Preservation

Standard guidelines were followed for the pretreatment of sampling materials, storage, and transportation of fish and shellfish [39]. A total of nine fish and shellfish samples, i.e., Bombay duck (*Harpadon nehereus*), mackerel (*Scomberomorus guttatus*), pomfret (*Pampus argenteus*), sea gobies (*Parachaeturichthys polynema*), croaker (*Johnius belangerii*), hilsa (*Tenualosa ilisha*), brown shrimp (*Metapenaeus monoceros*), squid (*Sthenoteuthis oualaniensis*), and crab (*Scylla olivacea*), were collected from the sampling site. The collected organisms were wrapped in aluminum foil and promptly stored in an ice box and transported to the Laboratory of Aquatic Biodiversity and Ecophysiology, Department of Fish Biology and Genetics, where they were preserved at −20°C for further assessment.

### 2.3. Extraction of MPs From Fish and Shellfish

The separation of MPs from fish and shellfish followed the protocols described by Ferguson et al. [40] and Karami et al. [41]. Measurement of the length and weight of each fish and shellfish sample was taken before proceeding, and the gill and gastrointestinal tract (GIT) of fish, as well as the whole body of shellfish, were dissected using a wooden tray, knife, scalpel, and scissors. The weight of the gill and GIT were taken by using temporary-labeled Petri dishes and then placed individually into prelabeled glass jars. Subsequently, 120 mL of 10% KOH (w/v) and 30 mL of 30% H_2_O_2_ were added to each jar and incubated at 60°C for 3 days to ensure complete digestion of the organic matter. Following the incubation, samples were filtered through Whatman GF/C glass filter paper (pore size: 1.2 μm, diameter: 47 mm) using a vacuum pump. Additionally, the filter papers were oven-dried at 30°C and then transferred to glass Petri dishes containing silica gel and wrapped with aluminum foil to avoid external contamination prior to microscopic examination.

### 2.4. Isolation, Characterization, and Enumeration of MPs

The filter papers were meticulously inspected at various magnifications (4x and 10x) using a stereomicroscope fitted with a digital camera from Carl Zeiss Microimaging GmbH (Germany), capturing images of the MPs for further analysis. During microscopic observation, a hot needle test was carried out to confirm MP particles when necessary [42]. According to Zhao et al. [43], the visual identification of different forms of MPs was conducted based on their physical characteristics and categorized as fiber, fragment, or microbead. The total number of MPs was estimated, and the types of MPs in each sample were calculated. Additionally, the color variations and size distribution of MP particles were noted using ImageJ v.1.54G software.

### 2.5. MP Polymer Analysis by Attenuated Total Reflection–Fourier Transform Infrared (ATR–FTIR)

The types of MP polymers were identified using ATR–FTIR spectroscopy (Spectrum TWO, L1600400, PerkinElmer, Germany) by retrieving the MPs from the filter paper of the extracted samples [10, 37]. The examined wavelength ranged from 4000 cm^−1^ to 500 cm^−1^, with a resolution interval of 2 cm^−1^. Before each measurement, the diamond crystal was cleaned with 70% isopropyl alcohol, and a blank reading was taken to ensure background accuracy. Filter papers containing MPs were placed onto the ATR crystal for infrared spectral analysis. Samples were pressed against the crystal with a minimum force of 80 N to ensure optimal contact and accurate spectral acquisition. Polymer identification was deemed reliable when the spectral match exhibited a minimum similarity of 80%–85%. The acquired spectra were compared with reference spectra reported by Jung et al. [44] to determine the polymer types, as summarized in Table S2.

### 2.6. Hazard and Ecological Risk Assessment

Most of the previous studies have employed various techniques, and indicators like MP concentration, polymer type, and toxicity to assess aquatic MP risks [45]. MP contamination in fish and shellfish collected from the Bay of Bengal was evaluated using the polymer hazard index (PHI) (equation (1)), as described by Nithin et al. [4] and Ranjani et al. [46]. This method incorporated established protocols for hazard evaluation, considering both the environmental durability and toxicity of various polymer types. The PHI was evaluated according to the following formula:

(1) $$\begin{array}{c} \text{PHI}=\sum P_{n}\times S_{n}, \end{array}$$

where *S*_*n*_ = polymer hazard score acquired from Lithner et al. [47] and *P*_*n*_ = the fraction of a particular plastic polymer. The chemical toxicity of different MP polymers is calculated according to the hazard scores (*S*_*n*_).

Alongside polymer types, MP concentration serves as a key indicator for assessing MP-related risks [45]. In this study, the pollution load index (PLI) (equation (2)) was calculated based on MP concentrations to determine contamination levels in fish and shellfish samples [48]:

(2) $$\begin{array}{c} {\text{CF}}_{i}=\frac{C_{i}}{C_{0}},\\ \text{PLI}=\sqrt{{CF}_{i}}, \end{array}$$

where contamination factor (CF) is the ratio of the MP abundance in fish and shellfish samples (*C*_*i*_) to the background MP concentration (*C*_0_). Here, the lowest MP abundance estimated in this study was considered as the background MP concentration (*C*_0_) [45]. Hazard and risk categories for PHI and PLI are shown in Table S1.

### 2.7. Human Health Exposure to MPs Assessed by Shellfish Consumption

Assessing human exposure to MPs is challenging due to their physicochemical diversity, which affects toxicity and bioavailability in living organisms, along with the lack of standardized methods for detecting them in environmental and biological samples [35]. Health risk in current research has been calculated by using two analytical approaches based on the dietary exposure of MPs by consuming shellfish [49]. The first estimation accounted for consumption as per the European Food Safety Authority (EFSA) recommendations for shellfish intake: 150 g per week for adults [50], while the other was based on the number of shellfish eaten by each individual [35]. The average annual shellfish consumption per person in Bangladesh is 1.35 kg [51, 52]. So, the average amount of MPs found in the shellfish was used to estimate the intake of MPs from shellfish for humans, based on EFSA (equations (3) and (4)) recommendations or DoF (equation (5)) data, in the following equations:

(3) $$\begin{array}{c} \text{MP intake by humans per week }\left(\frac{\text{MP items}}{\text{week}}\right)=\text{Average of MP items in shellfish }\left(\frac{\text{items}}{g}\right)\times \text{recommended shellfish consumption per week }\left(g\right), \end{array}$$

(4) $$\begin{array}{c} \text{MP intake by humans per year}\left(\frac{\text{MP items}}{\text{year}}\right)=\text{Average of MP items in shellfish}\left(\frac{\text{items}}{g}\right)\;\times \text{recommended shellfish consumption per week }\left(g\right)\times \text{Number of weeks per year}, \end{array}$$

(5) $$\begin{array}{c} \text{MP intake by humans per year per capita}\left(\frac{\text{MP items}}{\text{year}/\text{capita}}\right)=\text{Average of MP items in shellfish}\left(\frac{\text{items}}{g}\right)\times \text{intake of shellfish per year per capita in the selected country }\left(g\right). \end{array}$$

### 2.8. Quality Assurance and Quality Control

The quality control procedures in this study were carefully designed to prevent any contamination from the moment of sample collection until the conclusion of laboratory analysis. Before field sampling, all equipment was thoroughly cleaned, and nonsynthetic tools were documented to avert potential field contamination. In the laboratory, a designated area was allocated for the study to avoid cross-contamination, and samples were processed in a fume hood with restricted access. Lab personnel wore clean gloves, face masks, and cotton lab coats instead of synthetic clothing during the experiments. Glass and metal tools were favored over plastic to minimize contamination risks. All glass containers, tools, and instruments were subjected to multiple washings with detergent or glass cleaner and rinsed with ultrapure deionized water or 70% ethanol before use [53]. Similarly, reagents and distilled water were filtered through a 1 μm cellulose membrane before use [54]. Samples were promptly covered with aluminum foil when not in use to prevent contamination. Blank tests were carried out to assess potential contamination from the lab environment, and any identified minor contaminants were subtracted from the final results [55]. All prepared liquid samples were filtered through Whatman GF/C glass filter paper. These comprehensive quality control measures ensured the accuracy and dependability of the research findings by minimizing the risk of contamination at every stage.

### 2.9. Statistical Analysis

The geographic location of the sampling spot was described using ArcGIS Desktop (v.10.8). The initial organization and processing of all raw data were carried out using Microsoft Excel 365. All statistical analyses were completed using IBM SPSS (v.27). Before conducting one-way ANOVA at *p* < 0.05, data normality was assessed using the Shapiro–Wilk test. Additionally, a nonparametric Kruskal–Wallis test at *p* < 0.05 was performed when the data were found not to be normally distributed. MP abundance in the gill and GIT samples of different fish was compared by using the Kruskal–Wallis and Dunn's test, while one-way ANOVA and Tukey's HSD post hoc test were used for shellfish. The figure illustrating the identified polymers was generated using Origin Pro v.10.2.0.118.

## 3. Results

### 3.1. Identification of MP Types in Fish and Shellfish

**Figure 2:**
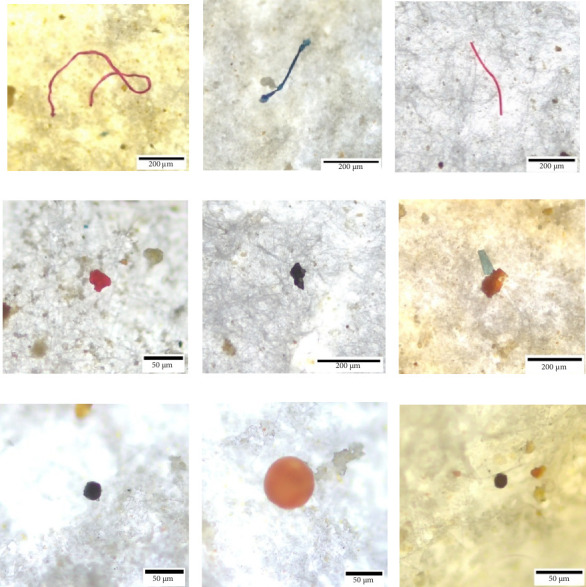
Microscopic image of some MPs directly taken from filter paper. Different shapes of fibers (a–c), fragments (d–f), and microbeads (g–i) were identified from different fish and shellfish samples.

The current study examined the presence of three common types of MPs—fibers, fragments, and microbeads—in the gills and GITs of fish, as well as in shellfish samples. Among these, fibers were the most abundant, characterized by their elongated, thread-like structure. These likely originate from synthetic textiles or fishing gear. Fragments, the second most prevalent type, were identified as irregularly shaped particles resulting from the breakdown of larger plastic debris. Microbeads, which appeared as small, smooth spheres, discs, or cylindrical particles, were the least frequently detected across all samples. These findings, illustrated in Figure 2, highlight the dominance of fibrous MPs in marine organisms and suggest varying sources and degradation pathways for different MP types.

### 3.2. Abundance of MPs and Shape Distribution in Gill and GIT of Fish

The concentrations of three MP types—fibers, fragments, and microbeads—were quantified in the gill and GIT of six fish species, with statistically significant differences (*p* < 0.05) summarized in Table 1. In gill tissues, fiber concentrations averaged 2.03 ± 0.22 items/g and varied significantly among species. Bombay duck exhibited the highest fiber levels, whereas pomfret showed significantly lower concentrations. Fragment concentrations in gills, averaging 1.46 ± 0.12 items/g, did not differ significantly across species. In contrast, microbead levels in gills (0.71 ± 0.11 items/g) showed significant interspecies variation (*p* < 0.05), with hilsa presenting notably higher concentrations than mackerel. In the GIT, both fiber (1.39 ± 0.20 items/g) and fragment (1.14 ± 0.19 items/g) concentrations were significantly elevated in hilsa (*p* < 0.05), while bombay duck had the lowest levels. Microbead concentrations in the GIT remained consistently low across all species (0.59 ± 0.08 items/g), with no statistically significant differences observed.

**Figure 3:**
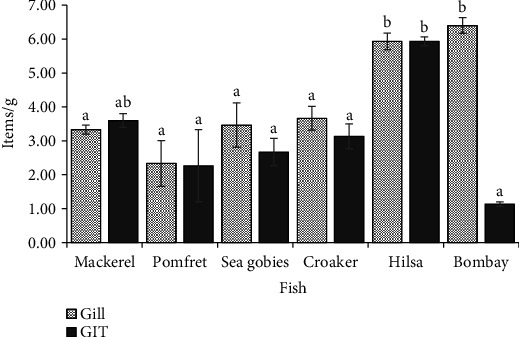
Mean abundance of MPs in the gill and GIT of the fish (values are means ± SEM. Different superscript letters differ significantly following nonparametric Kruskal–Wallis test at *p* < 0.05).

The total abundance of MPs across all the fish samples in the gill and GIT is demonstrated in Figure 3. In the case of gill, the highest abundance of MPs was recorded in the bombay duck, followed by hilsa, croaker, sea gobies, mackerel, and pomfret. In contrast, hilsa exhibited the highest level of MPs in the GIT, while the lowest was documented in bombay duck (Figure 3).

### 3.3. Abundance of MPs and Shape Distribution in Shellfish

**Figure 4:**
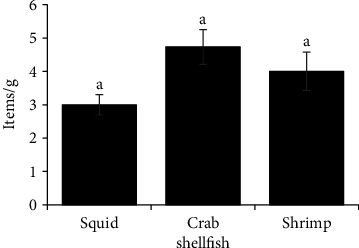
Mean abundance of MPs in shellfish (values are means ± SEM, a common superscript letter indicates no significant difference [*p* < 0.05] as analyzed by one-way ANOVA and Tukey's test).

In shellfish, no statistically significant differences were observed in the concentrations of MP types—fibers, fragments, and microbeads—across the examined species, as shown in Table 2. Among these MP types, fibers and fragments were consistently more abundant than microbeads in all species. Crabs exhibited the highest mean concentrations for each MP type, followed by brown shrimp and squid. This trend suggests a species-specific accumulation pattern, potentially influenced by habitat or feeding behavior. Furthermore, Figure 4 illustrates that the total MP abundance across the three shellfish species. Crabs had the highest concentration at 4.73 ± 0.520 items/g, while squid showed the lowest at 3.00 ± 0.305 items/g. These findings highlight the variability in MP accumulation among shellfish, despite the lack of statistically significant differences.

### 3.4. Size and Color Distribution of MP Type in Fish

**Figure 5:**
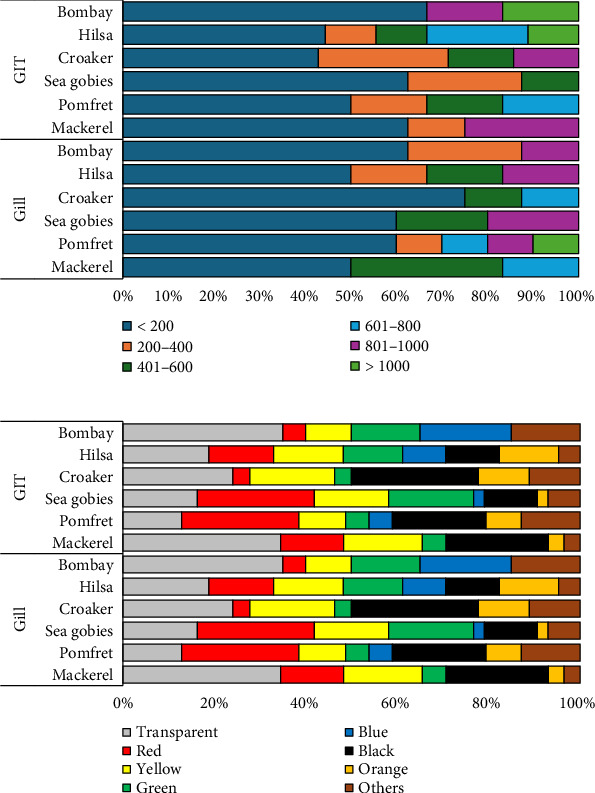
(a) Size and (b) color diversification of MPs in the gill and GIT of fish.

Figure 5(a) illustrates various sizes of MPs found in fish samples, categorized into six groups based on their sizes. The most prevalent group was MPs < 200 μm, making up 57% of the total, followed by those in the range of 200–400 μm (13%), 401–600 μm (10%), 801–1000 μm (9%), 601–800 μm (7%), and those larger than 1000 μm (3%). Compared to fragments and microbead-type MPs, the fiber type tended to be longer in fish samples. Nearly all fragment and microbeads-type MPs were smaller than 200 μm. The MPs in fish gill and GIT samples appeared in various colors, including transparent, red, yellow, green, blue, black, orange, and others, as depicted in Figure 5(b). The average color distribution of MPs in fish samples was ranked as follows: transparent > black > red > yellow > green > orange > others > blue. MPs of all different colors were detected in every fish sample.

### 3.5. Size and Color Distribution of MPs in Shellfish

**Figure 6:**
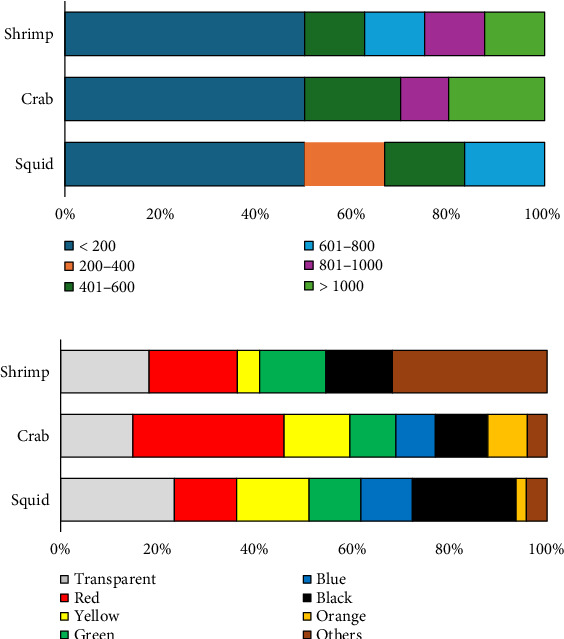
(a) Size and (b) color distribution of MPs in shellfish.

Figure 6(a) demonstrates various sizes of MPs found in shellfish, classified into six categories based on size. The most dominant group consisted of MPs < 200 μm (50% of total data), followed by those ranging from 200–400 μm (4%), 401–600 μm (17%), both 601–800 μm and 801–1000 μm (8%), and those exceeding 1000 μm (13%). Nearly, all MP types in shellfish (shrimp, crab, and squid) were smaller than 200 μm. The mean color distribution of MP type in shellfish samples was categorized as follows: red > transparent > black > yellow > green > blue > others > orange (Figure 6(b)).

### 3.6. MP Polymer Analysis by ATR–FTIR

The FTIR spectra of the MP particles were identified in the collected fish and shellfish samples. Specifically, MP particles were identified in the GIT and gills of various fish and the whole body of shellfish, such as the bombay duck, mackerel, pomfrets, sea gobies, croaker, hilsa, brown shrimp, squid, and crab of the Bay of Bengal. Ten polymers were detected from the FTIR spectra of 500 cm^−1^ to 4000 cm^−1^. The peak values were compared with the absorption bands for polymer identification. The identified polymers with their peak wavelength values are discussed in Table S2 and Figure S1. The identified polymers were polymethyl methacrylate (PMMA), ethylene vinyl acetate (EVA), nitrile butadiene rubber (NBR), polypropylene (PP), polycarbonate (PC), acrylonitrile butadiene styrene (ABS), nylon, high-density polyethylene (HDPE), polyvinyl chloride (PVC), and polyurethane (PUR). However, the polymer detection rate was 88.24% in this study. It has been noteworthy that the identified polymers comparatively showed higher density. The chemical composition of the fish and shellfish samples contained PMMA (43.33%), HDPE (1.67%), ABS (6.67%), NBR (1.67%), PC (3.33%), EVA (23.33%), PVC (6.67%), PUR (3.33%), nylon (5%), and PP (5%). PMMA, nylon, EVA, PUR, PVC, and PP were highly present in the GIT and gills of mackerel, hilsa, pomfret, and crab. In Figure S1, the wavenumber (cm^−1^) vs transmittance (%) graph indicates the particle's chemical bonds on the surface.

### 3.7. Hazard Risk Assessment by PHI

In this study, NBR and PP are categorized as low hazard risk (hazard category II) based on the PHI value, whereas HDPE is considered a medium hazard risk (hazard category III). Conversely, PMMA, ABS, PC, PVC, and PUR are classified as very high hazard risk (hazard category V), while EVA and nylon are categorized as high hazard risk (hazard category IV) (Table 3). Additional information on the hazard statements based on PHI and hazard levels can be found in Table 3.

### 3.8. Ecological and Health Risk Assessment

**Figure 7:**
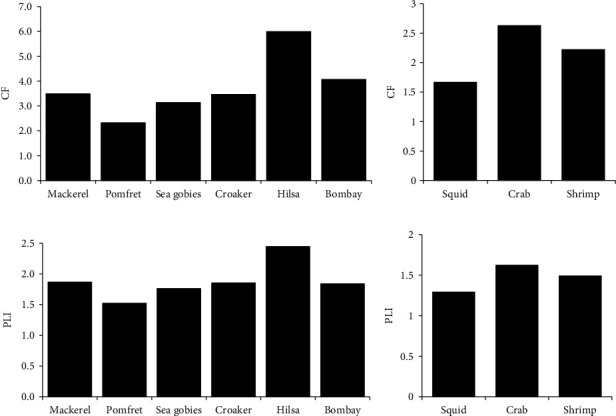
(a, c) CF and PLI of fish and (b, d) CF and PLI of shellfish.

The CF value of fish samples is described in Figure 7(a), where the highest amount of CF was in hilsa (5.99) and the lowest in pomfret (2.32). CF value was approximately the same in mackerel, sea gobies, and croaker except for the bombay duck. The CF value in shellfish is illustrated in Figure 7(b). The highest amount of CF was found in crabs (2.63), followed by shrimp (2.22), and squid (1.67). The PLI values of all fish samples were below 10, meaning a low-risk level, classified as risk category I. Figure 7(c) shows that the highest amount of PLI was in hilsa, followed by mackerel > croaker > bombay duck > sea gobies > pomfret. In the case of shellfish, the PLI value was also under 10, indicating low pollution by the MP type. Figure 7(d) illustrates that the highest amount of PLI was observed in crabs, and the lowest amount of PLI was in squid.

Consuming the fish and shellfish species examined in this study has the potential to induce toxicity in humans due to the presence of identified MPs. Therefore, the study estimated the weekly, annual, and yearly per capita intake of MPs for adults in Bangladesh. The average number of MPs in the combined muscle tissues of the 3 shellfish species in this study is 3.91 items/g. Based on the EFSA [50], overall, MP intake of shellfish by humans is 586.67 items per week and 30,506.67 items per year. On the other hand, 5280 items of MP are taken per year per capita, according to DoF [51], from shellfish consumption.

## 4. Discussion

MP contamination endangers the marine environment by exerting detrimental effects on marine life and disrupting entire ecosystems [56]. The vast river network in Bangladesh substantially transports plastic waste to the coast and sea, either directly or indirectly, through water runoff, wind dispersal, rainfall, and flooding [10]. A prior study suggested that approximately 61.3 × 10^9^ MP items are released into the Bay of Bengal daily through the complex river and estuarine system, highlighting an important environmental concern [57]. Recently, Mercy & Alam [58] and Hossain et al. [59] found that MPs are prevalent in water, sediment, coastal fish, and shellfish species in the Bay of Bengal, posing a health risk through increased toxic substance accumulation in those who consume contaminated seafood. Furthermore, Ghosh et al. [60] highlighted the ongoing interaction between marine organisms and MPs, emphasizing the alarming prevalence of MP ingestion in fish. The negative impacts of MPs on various organisms, such as phytoplankton, crustaceans, gastropods, and fish, are also widely recognized [61]. Consumption of fish from coastal and marine waters has become a prime route for MPs, exposing human beings [62].

The distinct dietary choices and feeding behaviors of fish result in varying MPs ingestion among different species, providing significant evidence of species-specific accumulation [63]. Higher trophic fish, due to their increased energy demands [62] and greater food intake capacity, are highly susceptible to ingesting MPs [58]. This research found the highest MP abundance in *T*. *ilisha*, a planktivorous fish, which belongs to the pelagic zone, as similar findings were documented by Fatema et al. [54]. Pelagic fish are vulnerable to ingesting MPs due to their feeding habits, which involve filtering water rich in these particles [64]. Besides, MPs are increasingly prevalent in benthic and demersal fish and shellfish species [62]. Previous research observed that *H. nehereus* had significant MP contamination, averaging 6.98 ± 6.73 items/g from Indian fishing harbors [65]. In the GIT of this fish from the Bay of Bengal, MP levels ranged from 25 to 198 items per fish, with a mean of 98.34 ± 53.11 items per fish [66]. Furthermore, Hossain et al. [67] found 8.72 ± 1.54 items/individuals in this species from the northern Bay of Bengal. Although the present study found *H. nehereus* displays high MP levels in gills, other fish species in similar environments exhibit varying contamination levels, influenced by factors like feeding strategies and habitat preferences. The feeding habits of *H. nehereus*, which involve filtering small particles from the water, might increase the abundance of MPs in the gill [68]. The rising prevalence of MPs in marine crabs, particularly in Brachyura species, has become a significant concern. Research has shown concerning levels of contamination, with average MP concentrations measuring 0.67 ± 0.62 items/g in crabs from Gujarat, India [69]. Brachyuran crabs are particularly vulnerable to MP accumulation due to their feeding behaviors, which often involve the consumption of contaminated prey [70]. *M. monoceros* is a bottom feeder and shows a higher accumulation of MPs than squids. The denser pollutants, which settle down in the bottom area, are consumed by the bottom feeders [71].  Table 4 shows metadata of MP contamination in fish and shellfish from previous studies.

In marine ecosystems, especially within the Bay of Bengal, various MP morphotypes, including fibers, fragments, microbeads, pellets, foams and filaments, have been observed previously [55, 72]. The current study examined the three most common MP types, i.e., fibers, fragments, and microbeads, in the Bay of Bengal, where fibers were predominant, followed by fragments and microbeads. Globally, fibers are the dominant form of MPs in marine environments, making up to 91% of the MP content and often outnumbering other particle types [73]. Previous research has constantly highlighted fibers as predominant in crustaceans [74]. Credible sources of fibers in the Bay of Bengal include textile fabrics, fishing nets, ropes, and lines [75], while fragments may come from larger plastic debris such as bottles and bags, break down into MPs through processes like photooxidation and mechanical wear [76]. Additionally, microbeads are derived from primary sources such as industrial ion media, cosmetics, or toiletries [62].

This investigation revealed that MPs < 200 μm made up approximately 57% of the total detected MPs among the fish samples, as well as in shellfish, indicating that around 50% of the identified MPs were under this size. Hossain et al. [67] confirmed the prevalent biopermeability of MPs < 500 μm in fish from the northern Bay of Bengal. This prevalence likely stemmed from the greater presence of smaller particles from the breakdown of larger plastics, weathering, or direct releases [77]. MP particles smaller than 200 μm are the most common in fish and shellfish [78]. This study found that transparent, black, and red MPs were the most common in fish and shellfish, aligning with previous research [79]. Lost fishing nets contribute to the transparent fibers after degradation [35]. Black and transparent MPs are widely used in packaging, plastic containers, beverages, and polybags, found extensively in the Bay of Bengal [58]. Erosion from vehicle tires releasing black fragments, plastic rubbish dumping and unmanaged waste dumps, and recycling plants disrupting debris are contributing to the presence of black and colorless fragments [80]. The predominantly detected polymers in ATR–FTIR are PMMA and EVA. Wires, packaging materials, and cables contribute to EVA polymers in aquatic environments [35]. Fibers originating from textiles, fishing activities, and other sources promote nylon, PMMA, PP, and ABS over natural aging [81]. PP and HDPE originated from extensively used commodities like plastic bottles, bottle tops, and toiletries bottles, highly found in fish [82].

The current study presents a potential threat to human health through PHI analysis of the found polymers from the organisms. Huang et al. [83] and Chatterjee et al. [84] developed PHI to evaluate the potential risks of different plastic polymers based on their chemical compositions. This study identified 10 different polymers, with only 3 falling into the low to medium hazard category, while the remaining were classified as high or very high hazard, which is similar to the findings of Siddique et al. [85]. Akter et al. [2] also assessed minor to medium PHI values in the collected fish from the southwestern region of Bangladesh. Furthermore, Nithin et al. [4] reported that PHI scores for fish from Parangipettai, located on the southeast coast of India, varied between 1 and 10, categorizing them within hazard II, which signifies a medium level of risk. The findings of this research strongly resonate with previous studies, emphasizing crucial levels of relevance and risk that cannot be ignored.

MPs typically enter the human body through two of the most common ways, via endocytosis and persorption [26]. Recent studies have identified the presence of MPs in human feces and human blood, indicating that humans can both absorb and excrete these particles [62]. Meanwhile, the objective of this study was to evaluate the possible risks associated with the consumption of fish and shellfish from the Bay of Bengal regarding MPs intake. CF and PLI are essential tools for assessing ecological risk in aquatic ecosystems [86]. In this study, CF values reflect that *T*. *ilisha* is more contaminated with MPs than other fish species, while crab has higher contamination levels compared to other shellfish. Similarly, the PLI values of the fish and shellfish samples in the present study were higher than 1 but lower than 10, indicating low to moderate risk of pollution from plastic debris. Jamal et al. [35] also estimated CF and PLI in fish of the northern Bay of Bengal and found similar findings, indicating MP contamination. The current findings also indicated that both CF and PLI varied among the species.

This rising hazard threatens public health, impacting the inhabitants of southwest Bangladesh, who depend on these commercially available species for protein. According to DoF [51], the per capita consumption of shellfish is 1.35 kg. About 5280 items of MPs are ingested by humans per year per capita by consuming shellfish. Based on EFSA [50], per week, shellfish consumption for an adult is 150 g. So, MP intake by an adult from the muscle tissue of shellfish is 586.67 items of MPs per week and 30,506.67 items per year. A study conducted by Cho et al. [87] reported that Koreans intake 212 MP particles annually via ingesting oysters, mussels, clams, and scallops. A previous study also reported that adults are projected to consume an average of 26,964 MP items per week, resulting in an estimated annual intake of 1,402,128 items; another estimate suggests an average yearly intake of approximately 1,244,460 MP items [35]. Considering the findings, it is essential to assess the toxic effects of MPs on the Bangladeshi population, as this has not been previously done. Future assessments should also include biodiversity and ecological susceptibility for a more comprehensive evaluation.

However, this study faced some limitations, the major one being that fish samples were not directly collected from the sea; fish had been purchased from the fishermen of the fishery ghat. Fishermen usually preserve fish in ice while harvesting, which may cause MP contamination. Also, the fishermen may use plastic containers and bags to carry the fish to the market. So, the time interval between harvest and market arrival poses a high risk of MP contamination. Besides these, while collecting the samples, trophic level, species age group, and seasonal variation were not considered. Thus, for better results, sediment and water for MP evidence need to be collected to make a complete overview of MP pollution in the Bay of Bengal. The study should be conducted with a large sample, focusing on all trophic levels for a longer period to evaluate the abundance of MPs over the different seasons with a more developed and broader human health impact evaluation.

## 5. Conclusion

This study investigated the occurrence of MPs in the gill and GIT of economically important marine fish species from the Bay of Bengal. Results revealed a significant accumulation of MPs across all sampled species, with notable variation in color, size, shape, and morphology, suggesting diverse sources and species-specific feeding behaviors. Polymer composition analysis and toxicity assessments further confirmed the presence of potentially hazardous MP types in these commercially valuable fish. However, a key limitation of the study was its exclusive focus on market-relevant species, omitting noncommercial species that may also play critical roles in the marine ecosystem. Future research should broaden its geographic and taxonomic scope to better understand the distribution and ecological consequences of MPs. Expanding this knowledge base is essential for developing targeted strategies to mitigate plastic pollution in the Bay of Bengal, thereby protecting marine biodiversity and ensuring the safety of seafood for both local communities and global consumers.

## Figures and Tables

**Table 1:** Abundance of MPs (items/g) in the gill and GIT of the fish.

Fish samples	MP type	Mackerel	Pomfret	Sea gobies	Croaker	Hilsa	Bombay duck	Total
Gill	Fiber	2.00 ± 0.31^a^	0.93 ± 0.29^a^	1.33 ± 0.18^a^	1.73 ± 0.18^a^	2.87 ± 0.37^ab^	3.27 ± 0.35^b^	2.03 ± 0.22
	Fragment	1.13 ± 0.24^a^	1.07 ± 0.24^a^	1.33 ± 0.41^a^	1.40 ± 0.23^a^	1.80 ± 0.31^a^	2.00 ± 0.12^a^	1.46 ± 0.12
	Microbeads	0.20 ± 0.12^a^	0.33 ± 0.18^a^	0.80 ± 0.12^a^	0.53 ± 0.18^a^	1.27 ± 0.24^b^	1.13 ± 0.18^ab^	0.71 ± 0.11

GIT	Fiber	2.13 ± 0.33^ab^	1.13 ± 0.55^a^	0.93 ± 0.08^a^	1.00 ± 0.00^a^	2.53 ± 0.24^b^	0.60 ± 0.12^a^	1.39 ± 0.20
	Fragment	0.93 ± 0.18^a^	0.80 ± 0.50^a^	1.13 ± 0.29^a^	1.27 ± 0.18^a^	2.47 ± 0.13^b^	0.27 ± 0.07^a^	1.14 ± 0.19
	Microbeads	0.53 ± 0.08^a^	0.33 ± 0.08^a^	0.60 ± 0.12^a^	0.87 ± 0.29^a^	0.93 ± 0.24^a^	0.27 ± 0.08^a^	0.59 ± 0.08

*Note:* Values are means ± SEM. Means in a row without a common superscript letter differ significantly nonparametric Kruskal–Wallis test at *p* < 0.05.

**Table 2:** Abundance of MPs (items/g) in shellfish.

Samples	MP type	Squid	Crab	Brown shrimp
Shellfish	Fiber	1.33 ± 0.18^a^	2.00 ± 0.31^a^	1.67 ± 0.33^a^
	Fragment	1.07 ± 0.08^a^	1.73 ± 0.24^a^	1.67 ± 0.33^a^
	Microbeads	0.60 ± 0.12^a^	1.00 ± 0.31^a^	0.67 ± 0.33^a^

*Note:* Values are means ± SEM. Means in a row without a common superscript letter differ significantly (*p* < 0.05) as analyzed by one-way ANOVA and Tukey's test.

**Table 3:** Polymer hazard index (PHI) of fish and shellfish.

Polymers	Specific polymer percentage (Pn)	Hazard score (Sn)	Polymer hazard index (PHI)	Hazard category	Risk category
PMMA	43.33	1021	44,239.93	V	Very high hazard
HDPE	1.67	11	18.37	III	Medium hazard
ABS	6.67	6552	43,701.84	V	Very high hazard
NBR	1.67	1	1.67	II	Low hazard
PC	3.33	1177	3919.41	V	Very high hazard
EVA	23.33	9	209.97	IV	High hazard
PVC	6.67	5001	33,356.67	V	Very high hazard
PUR	3.33	7384	24,588.72	V	Very high hazard
Nylon	5	63	315	IV	High hazard
PP	5	1	5	II	Low hazard

Abbreviations: ABS, acrylonitrile butadiene styrene; EVA, ethylene vinyl acetate; HDPE, high-density polyethylene; NBR, nitrile butadiene rubber; PC, polycarbonate; PMMA, polymethyl methacrylate; PP, polypropylene; PUR, polyurethane; PVC, polyvinyl chloride.

**Table 4:** Comparison of MPs (items/g) in different fish and shellfish with other studies.

Species	Mean abundance (items/g)		Digestion	Location	References
	Gill	GIT			
Bombay duck	6.40 ± 0.23	1.13 ± 0.07	10% KOH and 30% H_2_O_2_	Bay of Bengal, Bangladesh	Current study
	—	1	50 mL of 30% H_2_O_2_		[60]
	—	0.37 ± 0.10	200 mL of 30% H_2_O_2_		[67]
	—	0.93	25–30 mL of 30% H_2_O_2_	Mumbai coast, India	[28]
	6.53 ± 1.58	4.13 ± 0.69	KOH and H_2_O_2_	Bay of Bengal, Bangladesh	[33]
Hilsa	5.93 ± 0.24	5.93 ± 0.13	10% KOH and 30% H_2_O_2_		Current study
	2.36 ± 0.24	1.99 ± 0.35	H_2_O_2_		[33]
	—	0.77 ± 0.09	200–400 mL of 30% H_2_O_2_		[54]
Shrimp		4.0 ± 0.58	10% KOH and 30% H_2_O_2_		Current study
	—	3.87 ± 1.05	200 mL of 30% H_2_O_2_		[67]
		21.51	200–250 mL of 30% H_2_O_2_		[58]
Mackerel	3.33 ± 0.13	3.60 ± 0.20	10% KOH and 30% H_2_O_2_		Current study
	2.56 ± 0.73	0.84 ± 0.45	30% H_2_O_2_		[88]
Pomfret	2.33 ± 0.67	2.27 ± 1.07	10% KOH and 30% H_2_O_2_		Current study
	—	0.41	20 mL of 20% H_2_O_2_	Yogyakarta, Indonesia	[89]

Croaker	3.67 ± 0.35	3.13 ± 0.37	10% KOH and 30% H_2_O_2_	Bay of Bengal, Bangladesh	Current study
		0.7	25–30 mL of 30% H_2_O_2_	Mumbai Coast, India	[28]
	5.37 ± 0.92	3.92 ± 0.71	KOH and H_2_O_2_	Bay of Bengal, Bangladesh	[33]
Crab	—	4.73 ± 0.52	10% KOH and 30% H_2_O_2_		Current study
	—	29.33 ± 11.53	10% KOH and 30% H_2_O_2_	Southwest India	[90]

Squid	—	3.0 ± 0.30	10% KOH and 30% H_2_O_2_	Bay of Bengal, Bangladesh	Current study
	—	0.24 ± 0.36	10% KOH	Pacific Ocean, China	[91]

Sea gobies	3.47 ± 0.66	2.67 ± 0.40	10% KOH and 30% H_2_O_2_	Bay of Bengal, Bangladesh	Current study
	—	0.84 ± 0.44 MPs/fish	180 mL of 10% KOH and 20 mL of 30% H_2_O_2_	Sundarbans, India	[84]
	—	2.62 ± 1.21 MPs/fish	180 mL of 10% KOH and 20 mL of 30% H_2_O_2_		[84]

## Data Availability

Data will be available upon reasonable request to the corresponding authors.

## Supporting Information

Table S1: Hazard and risk categories for PHI and PLI.

Table S2: Identified polymers and their peak wavelengths (cm^−1^).

Figure S1: Representative FTIR spectra of identified MP polymers from the samples studied, PMMA, Nylon, EVA, NBR, PP, PC, ABS, HDPE, PVC, and PUR, respectively.
