# Supplementary material for: Microplastics in Certain Commercial Finfish and Shellfish From Cox's Bazar Fish Landing Center, Bangladesh: Evaluating Abundance and Risks
Source: Scientifica (Cairo). 2025 Jul 7;2025:9515482. doi: 10.1155/sci5/9515482 (PMC12259316; doi:10.1155/sci5/9515482)
Supplement: Supporting Information — Additional supporting information can be found online in the Supporting Information section. [file 9515482.f1.docx]

**Microplastics in certain commercial finfish and shellfish from Cox’s Bazar fish landing centre, Bangladesh: evaluating abundance and risks**

Md. Shahriar Ahmed ^a^, Md. Khalid Saifullah ^a, b^, Mst. Afifa Khatun ^c^, Ananya Chakraborty ^a^, Anika Tasnim ^b^, Upayan Anam ^a^, Mohammad Toha ^d^, Md. Kamruzzaman Munshi ^c^, Mohammad Amzad Hossain ^a, *^, Mohammed Mahbub Iqbal ^a, *^

**Supplemental materials**

TABLE S1: Hazard and risk categories for PHI and PLI.

| **PHI** | **Hazard category** | **PLI** | **Hazard category** | **Risk category** |
| --- | --- | --- | --- | --- |
| 0-1 | I | <10 | I | Very low hazard |
| 1-10 | II | - | - | Low hazard |
| 10-100 | III | 10-20 | II | Medium hazard |
| 100-1000 | IV | 20-30 | III | High hazard |
| >1000 | V | >30 | IV | Very high hazard |

TABLE S2: Identified polymers and their peak wavelengths (cm^-1^).

| Polymer name | Peak wavelength values (cm^-1^) | Reference |
| --- | --- | --- |
| Polymethyl methacrylate (PMMA) | 2992, 2949, 1721, 1433, 1386, 1238, 1189, 1141, 985, 964, 750 | [1] |
| High density polyethylene (HDPE) | 2915, 2845, 1472, 1462, 730, 717 | [2, 3] |
| Acrylonitrile butadiene styrene (ABS) | 2922, 1602,1494,1452, 966, 759, 698 | [1, 4] |
| Polypropylene (PP) | 2950, 2915, 2838, 1455, 1377, 1166, 997, 972, 840, 808 | [1, 5] |
| Nitrile (NBR) | 2917, 2849, 2237, 1605, 1440, 1360, 1197, 967 | [1, 4] |
| Polycarbonate (PC) | 1015, 1081, 1164, 1232, 1506, 1775, 3000 | [6] |
| Ethylene-vinyl acetate (EVA) | 2916, 2850, 1742,1466, 1371, 1234, 1025 | [1, 2, 7] |
| Polyurethane (PU) | 468, 673, 731, 773, 875, 979, 1018, 1072, 1120, 1269, 1410, 1462, 1504, 1579, 1645, 1725, 2972, | [8] |
| Polyvinyl chloride (PVC) | 1427, 1331, 1255, 1099, 966, 616 | [1, 4] |
| Nylon | 3298, 2932, 2858, 1634, 1538, 1464, 1372, 1274, 1199, 687 | [1, 3, 4] |
| Latex | 2960, 2920, 2855, 1167, 1447, 1376 | [5, 9, 10] |

| 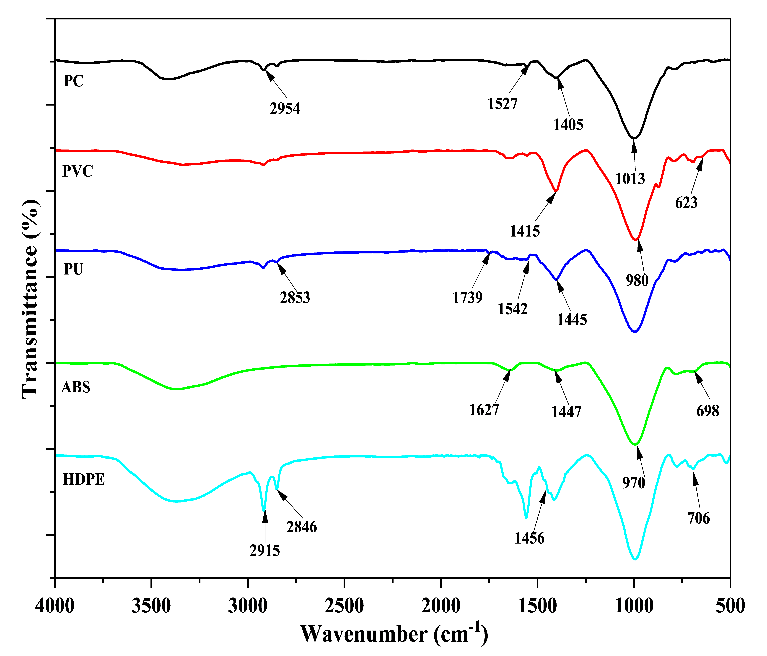 |
| --- |
| 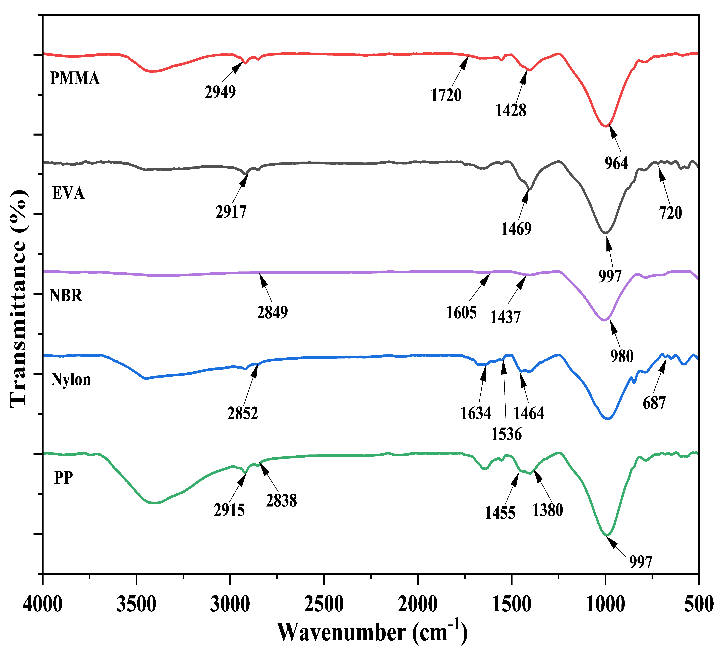 |

FIGURE S1: Representative FTIR spectra of identified MP polymers from the samples studied, PMMA, Nylon, EVA, NBR, PP, PC, ABS, HDPE, PVC, and PUR, respectively.

**References**

1. G.A.L. Verleye, N.P.G. Roeges, and M.O. De Moor: “Easy Identification of Plastics and Rubbers.” *iSmithers Rapra Publishing* , 2001.

2. H. Adelnia, H.C. Bidsorkhi, A.F. Ismail, and T. Matsuura: “Gas permeability and permselectivity properties of ethylene vinyl acetate/sepiolite mixed matrix membranes.” *Sep. Purif. Technol.* vol. 146, pp. 351–357, 2015.

3. I. Noda, A.E. Dowrey, J.L. Haynes, and C. Marcott: “Group Frequency Assignments for Major Infrared Bands Observed in Common Synthetic Polymers.” Physical Properties of Polymers Handbook. pp. 395–406. *Springer* (2007).

4. M.R. Jung, F.D. Horgen, S. V Orski, V. Rodriguez C., K.L. Beers, G.H. Balazs, T.T. Jones, T.M. Work, K.C. Brignac, S.J. Royer, K.D. Hyrenbach, B.A. Jensen, and J.M. Lynch: “Validation of ATR FT-IR to identify polymers of plastic marine debris, including those ingested by marine organisms.” *Mar. Pollut. Bull.* vol. 127, pp. 704–716, 2018.

5. J. Fang, Y. Xuan, and Q. Li: “Preparation of polystyrene spheres in different particle sizes and assembly of the PS colloidal crystals.” *Sci. China Technol. Sci.* vol. 53, no. 11, pp. 3088–3093, 2010.

6. A.M. Parshin, V.A. Gunyakov, V.Y. Zyryanov, and V.F. Shabanov: “Domain structures in nematic liquid crystals on a polycarbonate surface.” *Int. J. Mol. Sci.* vol. 14, no. 8, pp. 16303–16320, 2013.

7. R. Chércoles Asensio, M. San Andrés Moya, J.M. De La Roja, and M. Gómez: “Analytical characterization of polymers used in conservation and restoration by ATR-FTIR spectroscopy.” *Anal. Bioanal. Chem.* vol. 395, no. 7, pp. 2081–2096, 2009.

8. S. Bhattacharya, S.B. Chaudhari, S. Bhattacharya, and S. Chaudhari: “Study on Structural, Mechanical and Functional Properties of Polyester Silica Nanocomposite Fabric.” *Int. J. Pure Appl. Sci. Technol.* vol. 21, no. 1, pp. 43–52, 2014.

9. A.P. dos S. Pereira, M.H.P. da Silva, É.P. Lima, A. dos S. Paula, and F.J. Tommasini: “Processing and characterization of PET composites reinforced with geopolymer concrete waste.” *Mater. Res.* vol. 20, no. Suppl 2, pp. 411–420, 2017.

10. S.P. Tambe, S.K. Singh, M. Patri, and D. Kumar: “Ethylene vinyl acetate and ethylene vinyl alcohol copolymer for thermal spray coating application.” *Prog. Org. Coatings*. vol. 62, no. 4, pp. 382–386, 2008.
